# Comparison of Four RTK Receivers Operating in the Static and Dynamic Modes Using Measurement Robotic Arm

**DOI:** 10.3390/s21237794

**Published:** 2021-11-23

**Authors:** Jan Kadeřábek, Vadym Shapoval, Pavel Matějka, Milan Kroulík, František Kumhála

**Affiliations:** 1 Department of Agricultural Machines, Faculty of Engineering, Czech University of Life Sciences Prague, Kamýcká 129, 165 00 Prague, Czech Republic; shapoval@tf.czu.cz (V.S.); kroulik@tf.czu.cz (M.K.); kumhala@tf.czu.cz (F.K.); 2 Department of Technological Equipment of Buildings, Faculty of Engineering, Czech University of Life Sciences Prague, Kamýcká 129, 165 00 Prague, Czech Republic; matejkapavel@tf.czu.cz

**Keywords:** accuracy, precision, movement, dynamic properties, RTK, VRS, GNSS, INS, precision agriculture

## Abstract

While the existing research provides a wealth of information about the static properties of RTK receivers, less is known about their dynamic properties, although it is clear that the vast majority of field operations take place when the machine is moving. A new method using a MRA for the evaluation of RTK receivers in movement with a precise circular reference trajectory (*r* = 3 m) was proposed. This reference method was developed with the greatest possible emphasis on the positional, time and repeatable accuracy of ground truth. Four phases of the measurement scenario (static, acceleration, uniform movement and deceleration) were used in order to compare four different types of RTK receiver horizontal operation accuracy over three measurement days. The worst result of one of the receivers was measured at *SSR* = 13.767% in dynamic movement. Since the same “low-cost” receiver without an INS unit had *SSR* = 98.14% in previous static measurements, so it can be assumed that the motion had a very significant effect on the dynamic properties of this receiver. On the other hand, the best “high-end” receiver with an INS unit had *SSR* = 96.938% during the dynamic testing scenarios. The median values of the deviations were always better during uniform movements than during acceleration or braking. In general, the positioning accuracy was worse in the dynamic mode than in the static one for all the receivers. Error indicators (*RMS_err_* and *Me*) were found several times higher in the dynamic mode than in the static one. These facts should be considered in the future development of modern agricultural machinery and technology.

## 1. Introduction

The application of precision agriculture practices helps save operational costs and reduce the environmental burden, resulting in increased and improved food production while optimising the whole agricultural process [1]. Today, agriculture is confronted with a number of requirements, the introduction of which cannot be done without a significant modification of the current concept of production. A significant means of optimising inputs is the satellite navigation of agricultural machinery. In addition to time accuracy and enabling an optimised follow-up of individual work operations, more and more emphasis is placed on the level of accuracy of navigation devices. Most precision agriculture operations refer to the operation, guidance and control of machines to carry out agricultural tasks. The reliable and accurate positioning of these autonomous machines plays a key role in the set of operations comprising guidance, detection, action and mapping [2]. High accuracy and reliability will also be essential for the advent of robotic structures. Although people will continue to be involved in the decision-making process, it can be assumed that, with the growing intelligence of machines, their better interconnection and the accuracy of interventions, precision agriculture will become more and more automated. Furthermore, any deviation in accuracy at the beginning of the plant establishment process means introducing errors into all subsequent operations; in particular: increasing overlaps, higher costs, fuel consumption, seeds and chemicals and the carbon footprint. Confidence in the accuracy of RTK receiver positioning is essential in modern agriculture, as its reduction or even failure may negatively affect the benefits of the system. As described by the authors of reference [3], the benefits include the reduction of driver fatigue (guidance system reduces the effort required to maintain the correct machine route); increased productivity (allowing higher operating speed, faster headland turns and cost savings); cost reduction (there is a significant reduction in overlaps and omissions); reduced environmental impact; efficient overnight work; higher safety; better quality (the driver can focus on quality control) and improved ergonomics (reduction of stress of machine operators). Without precise positioning, autonomous navigation is dangerous for machine integrity and, more importantly, for workers.

Precise positioning can be assured by RTK methods that have already proved successful in a number of precise agriculture applications [1]. The possibility of measuring positions using the GNSS (Global Navigation Satellite System) laid the foundation for precise agricultural techniques. Combining the GNSS with RTK (Real-Time Kinematic) allows for a significant improvement of these methods [4,5]. Nowadays, the RTK method is one of the most accurate navigation systems available on the market, which can be used to obtain an absolute position anywhere on Earth’s surface with sufficient signal reception from GNSS satellites and a correction signal from a nearby reference station, called a base station [6,7]. RTK positioning can be widely used in different applications of precision agriculture, and it has already brought about positive effects.

Unmanned Aerial Vehicles (UAV) equipped with a camera can be useful in providing orthophotographs and improved resolution images. Ortho-mosaics can be generated based on images obtained from UAVs capable of flying over considerable large areas in a short time [8]. The georeferencing process constitutes one of the major challenges in the generation of ortho-mosaics. The authors of reference [9] suggested that, when the RTK method is applied on UAVs, positioning errors can be reduced and direct georeferencing ensured. In reference [10], an expensive RTK receiver placed on a drone was used to generate a detailed volumetric 3D model of plants. A drone with an RTK receiver was used to design the application of automatic transport to the exact location [11]. Very often, it relies on RTK receivers [12] to compensate for LiDAR locations in obtaining detailed point clouds. It is necessary to assume that the position deviation affects the distortion of the resulting point cloud objects composed of frames and, thus, limits the classification capability. LiDAR imaging is very fast and accurate. However, the limited position correctness of LiDAR for compensation during frame merging under large robot accelerations (e.g., UAV) requires very smooth and probably slow robot control.

RTK receivers to guide the tractor, possibly supplemented by the collection of data on land or crops, were described, for example, in references [4,13]; they make very efficient use of satellite signal corrections for autonomous tractor guidance and compare them with conventional correction reception. Cooperation between robots and humans can be defined at four points from the supervision of routine tasks, where robots perform a limited series of actions, to remote-controlled machines for dealing with unpredictable situations where a person is in the position of a passenger or supervisor [14]. The multi-robot tractor system in reference [15] relied on the coordinated guidance of the tractor fleet to the position obtained from the RTK receiver. In Farm Management Information Systems (FMIS), the RTK method can help increase the precision of tracking agricultural machines connected with other systems and, as a result, optimise the process chains, build smart farming systems and network various sets of data from diverse sources [16]. For most of the above applications, as well as countless others, a positioning accuracy equivalent to the accuracy of the acquired model or the precision of the intervention was required. All the above-mentioned studies used RTK receivers to obtain position information, but they assumed an error of its measurement almost always in the range from 0.01 m to 0.03 m.

### 1.1. Follow-Up on Previous Research

In the previous study of the research team of reference [17], the parameters of three RTK receivers during static measurements were investigated. The obtained results were compared with the findings of other studies [18,19]. It was confirmed that the *SSR* indicator had a significant effect on the positioning accuracy. This indicates the ability of the RTK receiver to reliably process the correction signal, which has a significant effect on the accuracy of positioning. It was concluded that the phenomenon of “false fixed solution” can occur for several reasons: the already mentioned erroneous estimation of phase ambiguities and the noise in the correction data from the base station, as well as the influence of the environment in which GNSS operates [20]. The evaluation of the *SSR* indicator was described in the study of reference [6], where it was clear that the distance of the base station from the receiver can affect the ability of the RTK receiver to resolve the ambiguity of the integer part of the carrier wave. The investigation confirmed the dependence on distance. In the study of the authors of reference [20], experimental rides with a robot following a predefined path were conducted, comparing two low-cost RTK receivers (both single-frequency multi-GNSS). These devices demonstrated a “fixed solution status” of 94% for the u-blox Neo-M8T RTK receiver (using open source RTK processing software named RTKLib) [21] compared to the result of 71.5% of RTK receiver SkyTra NS-HP. The experiment findings of the authors of reference [22] showed that, during measurements between buildings, the success of the “ambiguity resolution fixing rate” (referred to as *SSR*) was improved from 48.4% to 85.3%.

RTK receivers are more often used as reference systems providing positions or trajectories for other studies [7,12,23,24], a so-called ground truth. However, the major challenge is to ensure the real accuracy of the reference system (ground truth). The ground truth must be provided by a more precise method than using a verified system. In our opinion, ground truth can be considered as very reliable if it is determined in reference to one point anchored to the ground static measurement [17].

The authors’ study from reference [25] compared four types of RTK receivers also using SBAS, with one of the verified RTK receivers whose actual accuracy was burdened by the entire dynamic measurement result. Moreover, the dataset for evaluation here was significantly reduced to the selected 50 samples of each pairwise comparison. The authors of reference [22] obtained a reference trajectory using a self-developed dual-frequency high-precision postprocessing GNSS software receiver. The experiment results showed that, in the given test environment, the three-dimension precision of the relative positioning under open sky conditions was within 0.02 m. However, the study presented an evaluation of data from only two short periods of time (both approximately 6 min), where the movement speed of the measuring platform was given as approximately 5.5 m s^−1^ without specifying the definition of the acceleration performed. The evaluation of four RTK receivers against a postprocessed trajectory with a stated accuracy of 0.005 m was described by the authors of reference [26]. Among other things, this study provided specific values describing the accuracies depending on the artificially induced delay of the correction signal. The study presented an evaluation of the RMS error (*RMS_err_*) in the individual ENU components, where the horizontal error was no greater than 0.013 m in the fixed mode. The study defined a measurement scenario with a duration of 10 min but did not define the limiting speeds and acceleration of the measuring platform (only mentioned the specifications of the UAV used). Using the Oxford OxTs RT3002 reference system, the authors of reference [19] verified cheap versions of the RTK receivers, emphasising the complexity of evaluating their dynamic properties. The work specified measurements that took 14 min of driving a robot-driven vehicle on a test track. The results of the dynamic measurements were reported with an accuracy of 0.02 m but without specifying the metric used for this evaluation. No speed or acceleration constraint was specified in this work. The author of reference [27] compared GNSS/INS fusion (MEMS) against the reference trajectory of the Novatel SPAN-CPT RTK receiver. The measurements took 33 min on the road in traffic, with tunnel sections included, and evaluated the accuracy of the in-house developed fusion-based software with IMU and odometry. The study did not report comparable error rates, and, in addition, it was a method that did not evaluate the RTK receiver. The study of the authors of reference [28] used the ground truth values of the Imar INS device to evaluate the accuracy of the determination of the orientation of an object using the developed algorithms for UAV control. The dynamic measurements lasted 6 min without specifying the movements performed. The metrics were focused on angular variation, so it was not possible to provide a related indicator for any comparison. The study of reference [29] evaluated two low-cost RTK receivers based on the deviation of the distance of two antennas firmly anchored to a robot passing through the field moving at the speed 0.325 m s^−1^. A detailed comparable motion specification for the ground truth was not given. The stability of the positioning of these pairwise comparisons of distance deviations did not exceed 0.382 m. An interesting methodological idea for verifying the dynamic properties of the GNSS was described by the authors of reference [30]. A downhill rollercoaster with a set of photocells and antennas placed on a cart was used as a reference system. However, it focused on error indicators of instantaneous velocity deviations from samples of a mere 36 surveyed photocells. The measurements were made over a range of velocities from 12.5 m s^−1^ to 15.6 m s^−1^. A similar method was used by the study of the authors of reference [31], where a trajectory from a rail targeted by 32 points using the total station was used to evaluate GNSS receivers. Long-term measurements were made for 24 h at a constant speed of 4 m s^−1^. Within the resolution of the reference number points used, the distances performed and carrying platform speed limits, it was reasonable to use the proposed method only to evaluate the uniform movements. Unfortunately, the GNSS technology evaluated in this investigation was unsuitable for comparison of the RTK accuracy. A very similar investigation was carried out by the team of authors of reference [32] using an industrial three-axis robotic arm whose trajectory served as a very accurate ground truth. However, this study focused on the approach of evaluating the deviation angles (roll, pitch and yaw) for validating self-developed algorithms for the improvement of estimating accurately the attitude but did not present comprehensive deviation results and conclusions comparing multiple types of RTK receivers, such as this paper.

### 1.2. Objectives

As it is clear from the preview literature review, the most accurate possible navigation function is essential for the further development of modern agricultural technology (robotic structures, UAVs and others). Nevertheless, the existing research provides only minimal information on the influence of motion on the loss of accuracy, and the study of specific values of accuracy depending on motion is largely under-researched, although it is clear that the vast majority of field operations take place when the machine is moving, not when it is standing. The knowledge of navigation errors in the dynamic mode of operations is therefore essential for the further development of this technique.

Therefore, the main aim of this research was to investigate the effects of speed and acceleration on the properties of RTK receivers and compare the horizontal errors in the static and dynamic modes of operation. For these purposes, a new method was developed, based on a robotic MRA platform (Measurement Robotic Arm). The evaluation of four RTK receivers in this work was divided into the following subunits: the ability of the system to solve the problem of carrier phase ambiguities (*SSR*) (1), positioning the deviations of and their error indicators for the whole measurement period (2), positioning the deviations of and their error indicators for the period “FIX” (3), error indicators of the static measurements (4), recovery time of the “FIX” state after its loss (5), the linear dependence between the obtained series (6), the comparison of the positioning deviations according to the current state of the system (7) and the comparison of the positioning deviations according to the scenario phase (8).

## 2. Materials and Methods

First, it is appropriate to mention the technology principle fundamentals of RTK receivers. RTK receivers calculate the absolute position of the phase centre of a GNSS antenna, like GNSS receivers, determining the position point anywhere on Earth’s surface by estimating the pseudoranges from individual satellites moving in orbit [33]. As described in other papers [6,34,35], RTK uses observations to improve its positioning accuracy, which is doubly differentiated by a common pseudorange estimate and a correction signal. The correction signal is usually received from a stationary RTK receiver, called a “base station”. It is possible to use a dedicated base station [1,29] or to rent corrections from a base station of the built RTK correction network without the need to build a new one [26]. The base station must be located several kilometres from the RTK receiver. In some of the cases described in the literature [7,13], the desirable distance is up to 10 km or even 5 km, in some cases. The work of the authors of reference [17] described the Virtual reference Station (VRS) method based on the principle of a base station arbitrarily located in the vicinity of the surveyed area. The author of reference [7] compared VRS corrections with a common source of corrections. A more comprehensive pairwise comparison of the GNSS survey methods PPP and NRTK and static mode solutions was presented by the authors of reference [34]. There, the highest agreement was confirmed for the NRTK and static mode methods at the expense of the PPP method. This study reported only a relative accuracy relationship; a comprehensive statistical evaluation was lacking. The study focused only on the marginal deviations (minimum and maximum) in three directions. The work of the authors of reference [35] focused on a detailed investigation of the dependence of the carrier-to-noise ratio (C/N_0_) on the satellite elevation angle, base station baseline and frequency. One low-cost multifrequency RTK receiver u-blox ZED-F9P was used for verification, and it was confirmed that, even in static measurements, this receiver showed *SSR* ranging from 43.3% to 97.9%. This study also showed a twofold degradation in the vertical accuracy when replacing the fixed base station with an identical low-cost RTK receiver.

The use of so-called “moving base” technology was suggested as a suitable alternative for precision agriculture [36]. Scientific resources from the agricultural sector have not been devoted to this technology. Yet, a base station placed on a moving device could be used for corrections, allowing for the reduction of the baseline between the base and the rover.

In general, RTK positioning errors arise from the same causes as the GNSS. As described by Laud and Cross, the so-called multipath signal propagation can have a negative effect on a system’s accuracy, especially in environments with high obstacles obscuring the view of the orbit [37]. These and other typical reasons were described by the author of reference [23] and summarised by the collective authors of reference [38] as follows: the influence of signal transmission changes through the atmosphere (troposphere and ionosphere), deviations of ephemeris calculations (satellite trajectory), deviations of the satellite clock, construction properties and the location of the antenna. Especially, RTK receivers generally fail to solve the phase integer ambiguity of a signal for the measurement of the pseudorange. If they are able to solve this ambiguity, their measurements are precise [17].

In contrast to older technologies, today’s low-cost RTK receivers use only single-frequency and single-GNSS RTK methods [19]. As a result, the use of RTK receivers operating as multi-frequency and multi-GNSS allows for a successful estimation of the ambiguity. The authors of reference [39] compared a single-frequency RTK receiver using GPS with a combination of GPS + GLONASS and concluded with a positive result of the latter. The better visibility of satellites and their spatial arrangements have been described in previous studies using multi-GNSS receivers [40]. The authors of reference [41] showed significant findings in the ability to resolve the ambiguity using a combination of L1 and L2 ranges. Furthermore, this work positively evaluated the use of a Inertial Navigation System (INS) signal combined with RTK using the L1 frequency.

In practice, the high initial costs of multifrequency (also multi-GNSS) technology have been complemented with the widely used MEMS technologies (especially due to their favourable prices and often sufficient parameters) [42]. When a RTK receiver is unable to take measurements in the most accurate mode or, in the worst case, the GNSS signal fails completely, for example, due to the entrance into a tunnel, it is appropriate to use the so-called “Dead Reckoning” method [43]. This technique uses inertial position data from the AHRS systems from the IMU. With suitable algorithms allowing for the fusion of GNSS and RTK signals with data from AHRS, the INS is created, thus enabling the determination of the position with more accuracy. This system is capable not only of improving the ambiguity estimates but, also, in addition, it is equipped with spatial orientation mechanisms [21,27,28,44]. The authors of reference [45] described the significantly increased success rate of solving the ambiguity under a viaduct by the joint use of two RTKs of two mobile devices. This study also explored the possibilities to determine their relative position using LiDAR.

### 2.1. MRA Reference Trajectory

In a previous study [46], the development of a MRA reference platform was finalised, and the method for the measurements of RTK dynamic properties was verified. This methodology determined the process of data collection and data evaluation, aiming to obtain a precise reference trajectory. Following the previous study, it was possible to compare the reference trajectory to the measured trajectories of four selected RTK receivers and deliver, based on this comparison, specific values determining the properties of RTK receivers.

**Figure 1:**
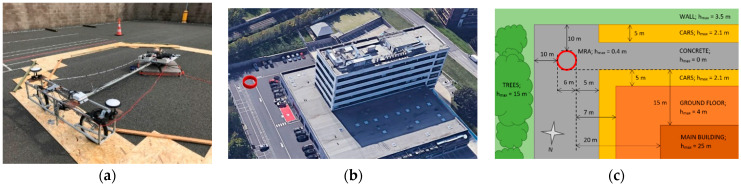
Recording the measurements from four RTK receivers in movement while performing evaluations based on the MRA method. The equipment performing the circular movement (**a**) was located on the horizontal surface near the building, trees, walls and personal vehicles (**b**). These obstacles obscuring the orbits were measured by an approximate method (**c**). The red circle determines the approximate trajectory of the MRA.

The measurements were performed in an outside test track of the development centre (Figure 1), which met the suitable conditions for measuring purposes. This place was selected based on its surface quality, necessary equipment and the possibility to perform recurring measurements at the same place during three measurement days. The measurements were performed on three days: 4 July 2019, 2 August 2019 and 28 August 2019.

**Figure 2:**
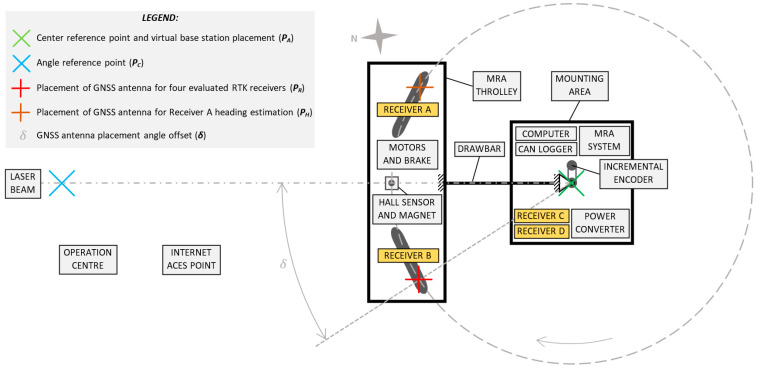
Drawing of the absolute alignment of MRA trajectory of two reference points, the placement of four evaluated RTK receivers, the instruments of MRA represented by blocks and the placement of the GNSS antennas.

The MRA was installed in a precisely marked location before each measurement day. This location was defined after the first day of measurement by accurately surveying the two reference points (Figure 2). For this surveying, Receiver A was used, which was the most credible. The catalogue accuracy of this device stated by the manufacturer was 0.01 m + 1 ppm. The static measurement, which was part of this study, then showed *RMS_err_* = 0.009 m (Evaluation 5). Both reference points were determined by the averaging of fixed positions with the same correction signal as the one used for the measurement. The first point defined the absolute location of the centre of the circular reference trajectory (*P_C_*), and the second point defined the initial absolute angle (azimuth) of this circular reference trajectory (*P_A_*). A beam of laser rangefinder Leica DISTO D810 representing a straight line came out from a tripod placed on *P_A_*, was aimed at the axis of rotation placed on *P_C_* and passed through the trolley peephole where the signal from the Hall sensor was detected. The distance between points *P_C_* and *P_A_* was 34 m.

The MRA was constructed with emphasis on the stiffness of the frame and designed so that the linkages allowed the smoothest and most stable movement of the trolley in the direction of a circular trajectory with a radius of 3 m. The following measures were taken to reduce the reference trajectory error due to the torsion of the structure:

- The robust construction of the rotation axis of the MRA was firmly anchored with screws and concrete blocks to the concrete surface in order to prevent possible deflection of the axis. Tilting the driven wheels in the direction of the tangent to the trajectory resulted in a reduction of the centrifugal force. The deflection of the rotation axis at the bearing height was measured in two perpendicular directions with a Hoteche HT284610 dial indicator located between the rotation axis rod wall below the bearing and the structure attached to concrete blocks at the maximum angle speed. These deflections were less than 2 mm in both directions.
- The vibration and possible tilting of the trolley in the pitch direction were eliminated by using sufficiently inflated tires with a wheelbase of 1.2 m and a very smooth and flat surface panelled with oriented strand board. Another aspect of reducing the trajectory error in this direction was the smooth acceleration (a_max_ = 0.7 m s^−2^) during the scenarios and the installation of the brake on the rear of the trolley. If, hypothetically, one of the sides of the trolley was raised to a height of 20 mm, this would mean a horizontal error (acting in the direction of the trajectory) less than 0.2 mm.
- The possible error caused by the torsion of the trolley and the deflection drawbar in the yaw direction was reduced by the tilting of the driven wheels in the direction of the tangent to the trajectory. This resulted in the elimination of wheel skidding and reduction of the centrifugal force. By placing the driven wheels and most of the weight (construction of the trolley and its components) at the end of the freely rotating lever, the possible deflection of the drawbar was eliminated. Another aspect minimising this possible deviation was the even distribution of the component weights on the trolley.
- The possible error caused by the torsion of the trolley frame and the deflection of the drawbar in the roll direction was minimised by the robust joint at the height of the trolley centroid.

Since the test track had a suitable tilt of the surface (less than 0.01 m to a radius of 3 m with respect to the circle created by the trolley wheels), the error according to the horizontal surface in the calculations of the location determination deviation was less than 0.02 mm.

The absolute angles were obtained based on the recorded times of microprocessor hours from the incoming leading edge of the three sensors: incremental encoder, a Hall probe and clock synchronisation signal Pulse per second (PPS) from the built-in MRA GNSS. Each signal was assigned a UTC time stamp in seconds per day in a microsecond resolution, as verified in the previous study of the authors of reference [45]. In contrast to the methodology used in the previous study, only one signal from the incremental encoder was used (the second signal was used only to determine the arm rotation direction). This decreased the MRA resolution from 5000 pulses to 2500 pulses related to one turn. The incremental encoder signal validity was algorithmically processed with the maximal set tolerance of ±3 redundant or missing pulses per turn. Measurement sections (turns bounded by two pulses from the Hall probe) failing to meet this condition were excluded. This ensured the accuracy of the angle distance (potential distance error in the direction of motion along the MRA reference trajectory) of 22.6 mm in relation to the MRA trajectory.

The obtained signal was further resampled using linear interpolation of the time-synchronous signal, corresponding to the clock of the given each RTK receiver. These samples were assigned corresponding coordinates in the ENU coordinate system with the same origin as the reference trajectory by subtracting the GNSS antenna angle offset (*δ*) of size 18°. Using the obtained data pairs, the values of instantaneous angular speed *v_r_* and instantaneous angular acceleration *a_r_* in relation to the trolley were calculated. Due to the high noise levels, these waveforms had to be smoothed out by a convolution filter with a core size equal to 7 for the instantaneous angular speed *v_f_* and 33 for the instantaneous angular acceleration *a_f_*.

On each measurement day, six sets of tests were performed, and the approximate angle speed of the arm (antenna of the RTK receivers) was set at the following values: 2 m s^−1^, 3.5 m s^−1^, 5 m s^−1^, 6.5 m s^−1^, 8 m s^−1^ and 9.5 m s^−1^. This represented the range of speeds used in agriculture, from soil tillage to transport. Each set of a concrete angle speed comprised three parts, forming the following scenario (Figure 8b): a gradual start from zero to the speed with the required value, even movements during the speed with the required value for 40 or 60 s approximately and breaking. For each speed, this approach was repeated at least four times.

### 2.2. Evaluated RTK Receivers

Four receivers were selected to be evaluated in this study: Receiver A, Receiver B, Receiver C and Receiver D. It is noteworthy that these selected devices differed in terms of their acquisition costs. The cheapest receiver, Receiver D, had an approximately seven times lower acquisition cost than Receiver C (both were delivered in pairs), an approximately fifteen times lower acquisition cost than Receiver B and more than thirty times lower acquisition cost than Receiver A. Table 1 summarises the essential properties of the selected RTK receivers.

Receiver A and Receiver B, containing an IMU unit, were located directly on the trolley. Receiver C and Receiver D were located on the mounting area near the centre of the MRA (yellow boxes in Figure 2). For the GNSS signal reception of the evaluated RTK receivers, one NovAtel GNSSS-850 antenna was used. The antenna (the phase centre of the antenna) was placed clockwise in the rear part of the trolley (*P_R_*). This signal was further divided to all RTK receivers using active splitter Antcom 4G1215S-XT4-P1. This solution made it possible that, to evaluate all of the four RTK receivers, one MRA reference trajectory was used. To determine the orientation (heading), Receiver A used the second antenna located in the front part of the trolley (*P_H_*). The heading was not evaluated in this study.

The computer ADLINK MXE-5400 served as the NTRIP client to receive the correction VRS signal from the provider geoorbit.cz. A virtual base station placement was located in the centre of the MRA rotation axis. The correction signal was distributed via the serial interface to all the RTK receivers. The power circuit of all the RTK receivers and the computer were galvanically separated from the MRA system power circuit.

The RTK receivers sent data in NMEA sentences in GGA format to the same computer, ADLINK MXE-5400, via another serial interface. The log periods for Receiver A, Receiver B and Receiver C were set to a frequency of 20 Hz. For Receiver D, the frequency was set to 5 Hz due to technical limitations. For each GGA sentence, the validity of the control check was verified, and the included geographical coordinates were converted to Cartesian coordinates of the ENU system with the selected start in the MRA rotation axis. A UTC time stamp in seconds of the days was assigned to each coordinate, including information on the status of solving of the carrier phase number ambiguity (system status).

### 2.3. System’s Ability to Solve the Problem of Carrier Phase Ambiguity (SSR)

Each sample from the obtained data contained status information from the NMEA sentence of a specific RTK receiver. The information could include one of the following three values: “FIX”—when the sample belonged to a period of a known integer value of the carrier phase, “FLOAT”—when the sample belonged to a period known only to the decimal of the carrier phase value and “GNSS”—when the sample belonged to a period when the RTK receiver was not able to process the correction signal. The *SSR* ratio, expressing the RTK ability to solve the problem of ambiguity of the integer part of the carrier phase, was calculated as follows:

System status ratio (*SSR*)—ability of the system to solve the problem of integer phase ambiguity (%):

(1) $$SSR=\frac{n_{f}}{n_{w}}*100,$$

where *n_f_*—dataset size of the samples with solved integer phase ambiguity (samples of the “FIX” state epoch) (-), and *n_w_*—dataset size of the measured samples (samples of the whole measurement) (-).

### 2.4. Positioning Deviations of and Their Error Indicators for the Whole Measurement Period

To determine the results, the deviation of the location determination was determined as the hypotenuse of a right-angle triangle, with perpendicular-based distance between the reference and measurement points in two mutually perpendicular directions (north and east):

Deviation (*d_i_*)—Euclidean distance between the reference and measured points (hypotenuse) on the horizontal plane (m):

(2) $$d_{i}=\sqrt{{\left(x_{ref}-x_{rtk}\right)}^{2}+{\left(y_{ref}-y_{rtk}\right)}^{2}},$$

where *x_ref_*—x-axis (east) coordinate of the reference point (m), *y_ref_*—y-axis (north) coordinate of the reference point (m), *x_rtk_*—x-axis (east) coordinate of the measured point (m) and *y_rtk_*—y-axis (North) coordinate of the measured point (m).

For the obtained range of deviations of the location determination, the error precision indicators were set using the following calculations:

Accuracy (*µ_err_*)—sample mean of deviations from the reference point (offset of error) (m):

(3) $${µ}_{err}=\frac{1}{n}\sum_{i=1}^{n}d_{i},$$

where *n*—dataset size of the measured samples (-) and *d_i_*—deviation from the reference point at the *i* index of a dataset (m).

Precision (*s_err_*)—sample standard deviation (stability of the positioning) (m):

(4) $$s_{err}=\sqrt{\frac{\sum_{i=1}^{n}{\left(d_{i-}{µ}_{err}\right)}^{2}}{n-1}},$$

where *n*—dataset size of the measured samples (-), *d_i_*—deviation from the reference point at the *i* index of a dataset (m) and *µ_err_*—sample mean of deviations from the reference point (accuracy) (m).

RMS error (*RMS_err_*)—value specified by the manufacturer (metric emphasising large errors) (m):

(5) $$RMS_{err}=\sqrt{\frac{1}{n}\sum_{i=1}^{n}d_{i}{}^{2}},$$

where *n*—dataset size of the measured samples (-) and *d_i_*—deviation from the reference point at the *i* index of a dataset (m).

To determine the indicators describing the properties of all the four RTK receivers (*µ_err_*, *s_err_* and *RMS_err_*), no method was used to remove the deviation outliers.

### 2.5. Positioning Deviations of and Their Error Indicators from the Period “FIX”

When only the samples marked as “FIX” in the NMEA messages (period when the RTK receiver was able to resolve the carrier phase ambiguity) were selected, the total set of measured coordinates that can be considered as accurate was obtained. For the evaluation, the same formulas were used to determine the error indicators (*µ_err_*, *σ_err_* and *RMS_err_*) similarly to the preceding Evaluation (2). The *RMS_err_* indicator from the “FIX” period was suitable as a value to be compared with the values in the manufacturers’ catalogues (Table 1).

### 2.6. Error Indicators of Static Measurement

For comparison purposes, static measurements of the RTK receivers were performed. During these measurements, the same conditions were maintained as in the dynamic measurements of the four RTK receivers: measuring environment, measuring period in three same days and setting of the RTK receivers, including reception of the correction signal and logging of NMEA messages. These static measurements were performed according to the methodology described in reference [17]. Receiver A was selected to determine the reference points by calculating the average coordinates from the “FIX” period of each of the three measuring days, such as in the dynamic one. The same error indicators (*µ_err_*, *σ_err_* and *RMS_err_*) were used to calculate the error indicators for the static measurements as for the dynamic measurements in Evaluation (2) and Evaluation (3). The *RMS_err_* indicator from the “FIX” period was suitable as a value to be compared with the values in the manufacturers’ catalogues (Table 1).

### 2.7. Recovery Time of “FIX” State after Its Loss

Due to the frequent observation of the state of inability of the RTK receivers to successfully process the correction signal (given the low *SSR* ratio), these periods were further investigated. These observations (*o*) were searched in both the static and dynamic measurements, and only those with known failure time and recovery time boundaries were used. Continuous logging of the NMEA messages between these boundaries was a necessary condition. The number and duration of these outages were evaluated:

- *n_o_*—number of detected outages from the “FIX” state (-),
- *min*(*t_o_*)—the shortest duration of the outage (s)
- *max*(*t_o_*)—the longest duration of the outage (s),
- *µ*(*t_o_*)—the mean duration of the outage (s) and
- *Me*(*t_o_*)—the median duration of the outage (s).

### 2.8. Linear Dependence between Obtained Series

Based on the Pearson correlation coefficient with a set value *α* = 0.05, the dependences between the obtained values of each RTK receiver were evaluated:

- *r_dv_*—correlation of the positioning deviations *d_i_* with the instantaneous angular speed of the trolley *v_i_*, and
- *r_da_*—correlation of the positioning deviations *d_i_* with the instantaneous angular acceleration of the trolley *a_i_*.

For correlations with the current RTK receiver system status, three possible statuses were evaluated with the following linear scale: “FIX” = 2, “FLOAT” = 1 and “GNSS” = 0:

- *r_sv_*—correlation of the current system status *s_i_* with the instantaneous angular speed *v_i_*,
- *r_sa_*—correlation of the current system status *s_i_* with the instantaneous angular acceleration *a_i_* and
- *r_sd_*—correlation of the current system status *s_i_* with the positioning deviations *d_i_*.

### 2.9. Comparison of Positioning Deviations according to the Current State of the System

The comparison of the whole deviation dataset of each RTK receiver between its three possible states (“FIX”, “FLOAT” and “GNSS”) was performed, using the median values from these datasets, as well as the presentation in Boxplot. The expression of the error using the median value Me was not burdened by the error of outliers as much as the expression of the error by *RMS_err_*. This presentation depicted the distribution of the deviations in four quartiles, where the median of the deviations was located between the boundaries of the two inner quartiles. From both boundary quartiles, the outliers were removed per the recommended value *k* = 1.5 per study [47], which corresponded to the designation of 0.7% total as the potential outliers where a normal distribution was assumed. The Boxplot function was used from the matplotlib library in Python.

### 2.10. Comparison of Positioning Deviations according to the Scenario Phase

The whole dataset was also compared in terms of its current scenario phase. Four phases of the scenario were identified: static (“STAT”)—when the trolley did not move, acceleration (“ACC”)—when the trolley arm was gradually accelerated from a zero angular speed to the required angular speed, uniform movement (“UNIF”)—when the trolley arm performed a uniform movement at the required angular speed and deceleration (“DEC”)—when the trolley arm was gradually braked to a zero angular speed. Individual samples of the dynamical measurements were divided per value of instantaneous angular acceleration between three ranges: for “ACC” in the interval (−∞; −0.2 > m s^−2^, for “UNIF” in the interval (−0.2; 0.4 > m s^−2^ and for “DEC“ in the interval (0.4; ∞) m s^−2^. For the “STAT” scenario phase, a separate set identical to that in section Evaluation (4) was used. As in the preceding case of Evaluation (7), this evaluation was performed based on the median values from the datasets and presented in Boxplot. These evaluations were also performed separately for the range of deviations for the whole measurement period (“FIX” + “FLOAT” + “GNSS”) and separately for the subset of the samples from the period “FIX”.

## 3. Results and Discussion

### 3.1. Ability of the System to Solve the Problem of Carrier Phases Ambiguity (SSR)

Table 2 shows the number of samples *n_w_* for each RTK receiver from the entire measurement period to which a valid MRA reference coordinate could be assigned. The number of *n_f_* samples that were marked as “FIX” in the NMEA report was also given. Furthermore, the calculated *SSR* indicator was given, representing the ability of the RTK receiver to solve the ambiguity of the phase of the carrier wave during the entire measurement period. According to the values obtained during the dynamic measurements, it was evident that the worst result was achieved by the cheap Receiver D with the *SSR* value of 13.8%. In contrast, during the static measurements in a previous study [17], Receiver D surprisingly proved to be the best, with an *SSR* value of 98.1%. In these dynamic measurements, Receiver A reached the best *SSR* value, as expected, with 96.9%.

Based on these results, it could be concluded that Receiver D was more suitable for use at very low speeds, because during the dynamic motion, it was not able to determine the integer value of the carrier phase as well as with more expensive receivers (most of the time, it occurred in the “FLOAT” or “GNSS” states), as well as RTK receivers with a higher purchase price. At the same time, it was clear that Receiver A and Receiver B were best able to maintain this condition for accurate measurements even during movement. Since both of these localisation systems used an RTK receiver with a built-in INS (Inertial Measurement System), it could be assumed that this feature was supported by the fusion with the INS that the receivers were equipped with. This hypothesis was also proven by reference [48], whose research suggested a worse accuracy of RTK receivers without an INS unit than RTK receivers with an INS unit.

### 3.2. Positioning Deviations of and Their Error Indicators for the Whole Measurement Period

**Figure 3:**
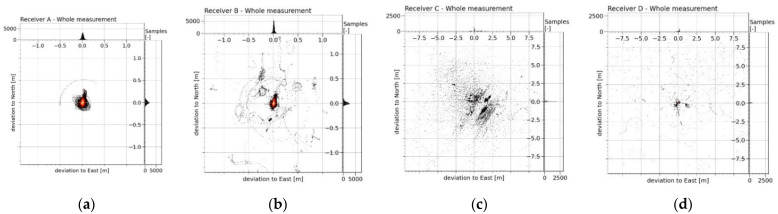
The density of occurrence of the horizontal deviations from the reference trajectory of the MRA in the latitude and longitude of four RTK receivers (**a**–**d**) from the whole measurement period. The horizontal distribution of the deviations was plotted in the graph of each RTK receiver in the large square area at the bottom-left. The percentage ratio of the occurrence density of the deviation in the area was plotted using a colour scale in a linear normalised distribution (where: black = 0%, red = 50% and white = 100%). The narrow bar histograms on the right and top show the density of the occurrence of the deviation in latitude and longitude in units of the obtained samples. The density of all three views of each image was calculated for Receiver A and Receiver B (**a**,**b**) from the displayed range (−1.5 m to 1.5 m) in the number of 1500 bins (resolution: 2 mm). Due to the large variance of the RTK deviations of Receiver C and Receiver D (**c**,**d**), the approximation of the displayed area of the graphs was adjusted. For these receivers, the density was calculated from the displayed range (−10 m to 10 m) in the number of 6500 bins (resolution: approximately 3.1 mm).

**Figure 4:**
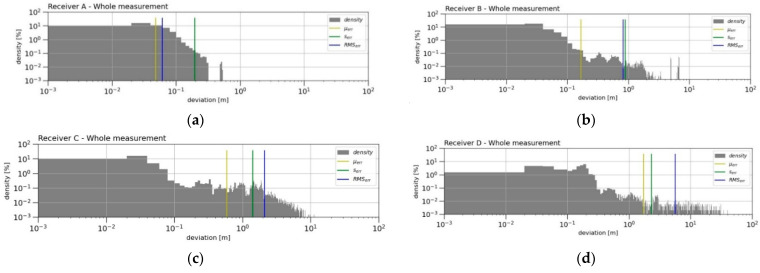
The density of the deviations in the graphs of all the RTK receivers (**a**–**d**), together with the calculated error indicators *µ_err_*, *σ_err_* and *RMS_err_* from the whole measurement period. The histogram uses a scale of logarithm base 10 to plot both axes. The density of occurrence of the deviation values was calculated identically for all the four receivers from the range (0–40 m) in the number of 2000 bins (resolution 20 mm).

The graph (Figure 3) plots histograms of the density of the horizontal deviations from the MRA reference trajectory in latitude and longitude (north and east). The occurrence density of the deviations *d_i_* is further shown in Figure 4, together with the calculated error indicators (*µ_err_*, *s_err_* and *RMS_err_*).

In the whole measurement period, based on the submitted error indicators *µ_err_*, *s_err_* and *RMS_err_*, RTK Receiver A was undoubtedly evaluated as the best. The plotted density of the occurrence values of the positioning deviations (Figure 4a) showed that Receiver A did not show larger deviations greater than 0.5 m at all, while this maximum positioning deviation manifested itself only during one short section of the measurements (approximately 0.02% of the total measurement time). Apart from this nuance, Receiver A had the worst positioning deviation of 0.31 m. Receiver B displayed slightly worse accuracy indicators (Figure 4b). The largest error was reflected in the *RMS_err_* indicators, where this error was mainly influenced by remote values of the deviations (the maximum deviation was detected to be 6.9 m). The occurrence density and deviation distance were even more pronounced in the *RMS_err_* error of Receiver C (Figure 4c). The accuracy *µ_err_* and precision *s_err_* indicators for this receiver were already very large compared to the previous ones. The low resolution and the boundary of the graphs did not allow displaying the situation when the positioning deviation of this receiver gradually increased from a deviation of 10 m to higher values. This situation occurred twice during the measurements, and in the worst case, this exponential drift of the deviation stopped at 426.2 m. For these three mentioned receivers (Receiver A, Receiver B and Receiver C) most of the measured positioning deviations occurred in the area of centimetre units (positioning deviations up to 0.04 m had a more than 20% occurrence rate in all the measurements, while the occurrence of larger deviations had a gradually declining trend in the density of the values). However, the same cannot be said about the results of Receiver D (Figure 4d). The highest occurrence density of approximately 6% was found to be around 0.2 m of the positioning deviation. From a value of 0.3 m, the density distribution at that receiver was much flatter, and in one case, the positioning deviation drifted down to a value of 46.5 m. The calculated accuracy indicators (*µ_err_*, *s_err_* and *RMS_err_*) were the highest, which means that this receiver had, by far, the worst results in all the measurements. The *RMS_err_* value of this RTK receiver was almost 94 times higher than the best result of Receiver A.

### 3.3. Deviations of Positioning and Their Error Indicators from the Period “FIX”

**Figure 5:**
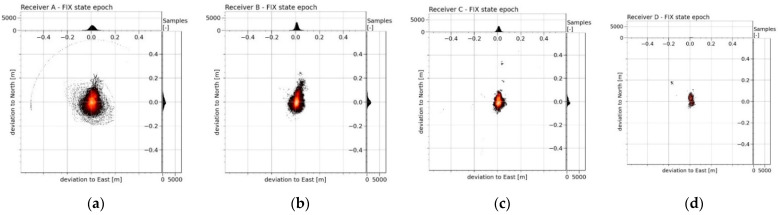
The density of occurrence of the horizontal deviations from the reference trajectory of the MRA in the latitude and longitude of the four RTK receivers (**a**–**d**) from the measurement period “FIX”. The horizontal distribution of the deviations was plotted for the graph of each RTK receiver in the large square area at the bottom-left. The percentage ratio of the occurrence density of the deviation in the area was plotted using a colour scale in a linear normalised distribution (where: black = 0%, red = 50% and white = 100%). Furthermore, the narrow bar histograms on the right and top show the density of the occurrence of the deviation in latitude and longitude in units of the obtained samples. The density of all three views of each image was calculated identically for all the four receivers from the displayed range (−0.6 m to 0.6 m) in the number of 1000 bins (resolution 1.2 mm).

**Figure 6:**
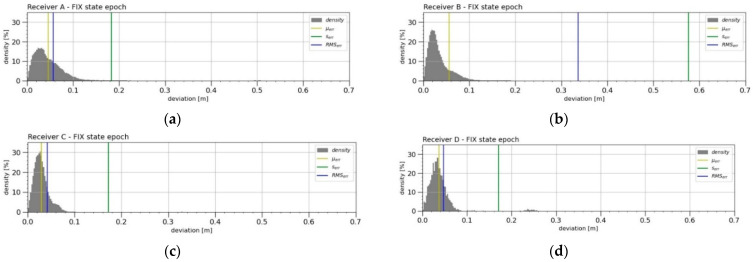
The density of deviations in the graphs of the four RTK receivers (**a**–**d**), together with the calculated error indicators *µ_err_*, *σ_err_* and *RMS_err_* from the measurement period “FIX”. The density of the values was calculated identically for all the four receivers from the range (0–5 m) in the number of 2000 bins (resolution 2.5 mm).

Another evaluation was performed on deviations from the period when the RTK receivers knew the integer value of the carrier phase (when in the “FIX” system state) and were measured in their most accurate mode. The graph (Figure 5) plots histograms of the density of the horizontal deviations from the MRA reference trajectory in latitude and longitude (north and east). The density of the deviations *d_i_* from this period is further shown in Figure 6, together with the calculated error indicators (*µ_err_*, *s_err_* and *RMS_err_*).

The evaluation was largely influenced by the size of the *n_f_* datasets. The size of the datasets was mainly affected by the *SSR* ratio of each of the receivers, as well as by the fact that Receiver D allowed samples with only four times lower frequency (5 Hz compared to 20 Hz). It must be noted that Receiver B also had a “failure” of the measurement section in these measurements, which is not shown in this view. This anomaly was also taken into account in the evaluation.

The Receiver A error indicators were calculated from the largest dataset and showed worse values than Receiver C (worst performing in the previous evaluation) and Receiver D. Receiver A was also loaded with a positioning error that occurred immediately from the initial start-up of the first measuring set (specifically at speeds of 0.1–3.7 m s^−1^ and an acceleration of 0.3–1.3 m s^−2^), when 98 samples showed a deviation of 0.45–0.54 m. Receiver B produced the worst result, as, during the period of one of the trolley starts (specifically at speeds of 0.1–7.9 m s^−1^ and an acceleration of −0.2–1.2 m s^−2^), it measured a deviation of 4.9–5.1 m in 436 samples. Paradoxically, the smallest error indicators were displayed by Receiver C (the second-worst in the previous evaluation). Its largest deviations ranged from 0.1 m to 2.6 m in the number of 21 samples occurring in two periods at high speeds (specifically, at speeds of 7.9–8.4 m s^−1^ and an acceleration of 0.6–2.6 m s^−2^). Receiver D had the least bulky dataset for this evaluation and good results of its error indicators. Its largest deviations ranging from 0.22 m to 0.26 m in the number of 50 samples and also occurred at higher speeds (specifically, at speeds of 6.6–6.8 m s^−1^ and an acceleration of 0.2 m s^−2^ to 0.3 m s^−2^).

The monitored error indicators *µ_err_*, *s_err_* and *RMS_err_* from the “FIX” state of the receiver did not equally reach large values compared to the deviations calculated from all the measurements. Large deviations were the most pronounced for Receiver D, which had the lowest *SSR* indicator. It can be concluded that, when the cheap RTK receiver can reach the “FIX” state, its measurement achieves a great accuracy, even greater than Receiver A. The results from a previous study [17] and the static measurement results of other authors [48,49] showed *RMS_err_* values of approximately 0.01 m. Another source [24] reported dynamic *RMS_err_* values between 3.4 mm and 3.8 mm during their dynamic measurements. However, the latter calculated the reported accuracy based on an unreliable trajectory from the reference system obtained through odometry by a field robot. It was assumed that the results of the error indicators measured by in this study influenced the dynamic scenarios serving as the basis of the study. After comparing the *RMS_err_* values specified by the manufacturer for the “FIX” system status, it was impossible to demonstrate that the RTK receivers met the stated values. The dynamic measurements were believed to be the cause.

### 3.4. Error Indicators of Static Measurement

The static measurements (Table 3) involved a smaller set of samples than the dynamic measurements (Table 2), but the entire recording of the three days together was approximately one hour and twenty minutes.

The stable unchanged position was mainly reflected in the ability to solve the ambiguity of the carrier phase (*SSR*). Receiver B and Receiver C were successful throughout all the measurements, and Receiver A was not able to resolve the ambiguity in one short period of three seconds. Receiver D was able to solve the phase ambiguity of the carrier wave in a static measurement almost three times more often than in a dynamic measurement. Nevertheless, compared to the results of a previous study [17], this receiver achieved an even worse result, which indicates that the established measurement conditions were not ideal for this low-cost RTK receiver.

The *SSR* result was reflected in the positioning indicators (*µ_err_*, *σ_err_* and *RMS_err_*) from the whole measurement period, while these errors were still smaller compared to the dynamic measurements. The error indicators determined based on the selection of samples from the “FIX” period showed smaller errors than in the dynamic measurements. A comparison of the resulting *RMS_err_* values with the catalogue values specified by the manufacturers revealed that Receiver A (*RMS_err_* = 0.01 m + 1 ppm) and Receiver D (*RMS_err_* = 0.025 m + 1 ppm) met the stated values. Receiver B and Receiver C did not narrow the stated values (*RMS_err_* = 0.01 m + 1 ppm).

### 3.5. Recovery Time of “FIX” State after Its Loss

Outages from the “FIX” state, which had a known period between its boundaries (outage and recovery), were not detected during the static measurements. The detected periods of outages came only from the dynamic measurements, which are presented in Table 4. Although Receiver A had the most outages, in most cases, it was able to recover back to the “FIX” mode within 2 s. In the case of two detected periods, however, the outages lasted 98 and 186 s. Receiver B was able to repair most outages within 3.4 s, but nine outages lasted in the range of 10–54 s, and one outage lasted even 228.3 s. The recovery of Receiver C was much worse, as twelve outages lasted between 13.45 and 88.35 s, and the longest outages lasted 118.8, 189.7, 236.1 and 753.15 s. Receiver D did not have as long of outages as Receiver C. Its longest outages lasted 146.4, 152.0, 230.4, 244.2, 286.2 and 472 s. Receiver D was able to repair most of the outages after 21.4 s, and thirty-six outages lasted between 10 and 91.6 s.

Based on the results above, it was possibly assumed that the RTK receivers with an INS unit (Receiver A and Receiver B) were able to return to the “FIX” state earlier than the receivers without an INS unit (Receiver C and Receiver D).

### 3.6. Linear Dependence between the Obtained Series

Table 5 summarises the linear correlation relationships of the instantaneous values of the RTK receivers expressed by the Pearson’s correlation coefficient. The correlations of the deviation of the positioning on the speed *r_dv_* or on the acceleration *r_da_* from the period of all the measurements did not have a significant effect. The dependencies calculated the same way in the evaluation from the “FIX” period were even weaker in significance than from the whole measurement period. No statistically significant linear relationship was found between any of the examined series. The evaluation of the dependence of the current system state on the speed *r_sv_* and the dependence of the current system state on the deviation *r_sd_* at Receiver C showed a very weak negative dependence. However, due to the proximity of the coefficient values to zero, even these dependencies could not be considered statistically significant.

### 3.7. Comparison of Positioning Deviations according to the Current State of the System

**Figure 7:**
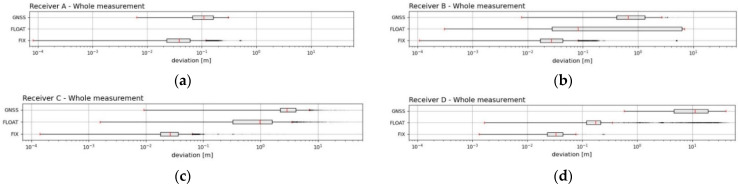
The distribution of the deviations during current states of the four RTK receivers from the whole measurement period (**a**–**d**). The boxplots display plots for each condition in a rectangular area ±25% of the values occurring around the median, as shown by the red line. The extreme red lines show the boundaries dividing the contents of the external observations from the outlier area. The outsiders are shown with a black dot with a set transparency of 20%. A logarithm of base 10 was used to display the deviation axis. For all RTK receivers, the current state of the system had a significant effect on their positioning deviations.

The graphs (Figure 7) below show the distribution of the positioning deviations during the individual states of the RTK receiver system in the form of boxplots. The values of the median deviations of the positioning can also be found in Table 6.

The first graph (Figure 7a) clearly indicates that only Receiver A reached the states “FIX” and “GNSS” during the whole measurement period. The value of its median from the “FIX” period *Me*(*FIX*) was the largest value of all of the four RTK receivers. Receiver B and Receiver D had the smallest median errors. Receiver B had the smallest value of *Me*(*FLOAT*), and Receiver C had a much higher value. In the “GNSS” period, Receiver A had the lowest *Me*(*GNSS*) value, and Receiver D had more than 101 times the *Me*(*GNSS*) value. It is evident that, for all the four RTK receivers, the deviations calculated from the median increased if the RTK receiver was in different states of the system in the following order of states: “FIX”, “FLOAT” and “GNSS”. From the median point of view, it could be assumed that, for all the RTK receivers, the positioning deviations were significantly influenced by the current state of the system.

### 3.8. Comparison of Positioning Deviations according to the Scenario Phase

**Figure 8:**
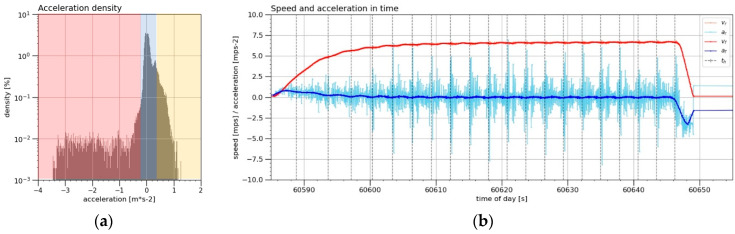
The distribution of the density of occurrence of the smoothed instantaneous angular acceleration *a_f_* values (s) from the whole measurement in a percentage ratio (**a**) with marking of the areas of the phases of the scenario, where “ACC”—yellow area in the interval of (−∞; −0.2 > m s^−2^, “UNIF”—blue area in the interval of (−0.2; 0.4 > m s^−2^ and “DEC”—red area in the interval of (0.4; ∞) m s^−2^. (**b**) The selected scenario (second day, approximate required speed 6.5 m s^−1^) plot *a_f_* values in the time of day (s), together with the other values: raw instantaneous angular acceleration *a_r_* (m s^−2^), raw instantaneous angular speed *v_r_* (m s^−1^), smoothed instantaneous angular speed *v_f_* (m s^−1^) and time of received signal from the Hall sensor *t_h_* (s).

Figure 8a displays the distribution of the density of occurrence of the values of *a_f_* from all the dynamic measurements in a percentage ratio, as well as three areas of selected scenario phases (“ACC”—yellow, “UNIF”—blue and “DEC”—red). Figure 8b aims to give a better idea of such division, displaying the current value of the acceleration *a_f_* (together with other values: *a_r_*, *v_f_*, *v_r_* and *t_h_*) of a selected scenario (second day, approximate required speed 6.5 m s^−1^). For the phases of the “STAT” scenario, a separate set obtained from the static measurements described in the section Evaluation (4) was used.

**Figure 9:**
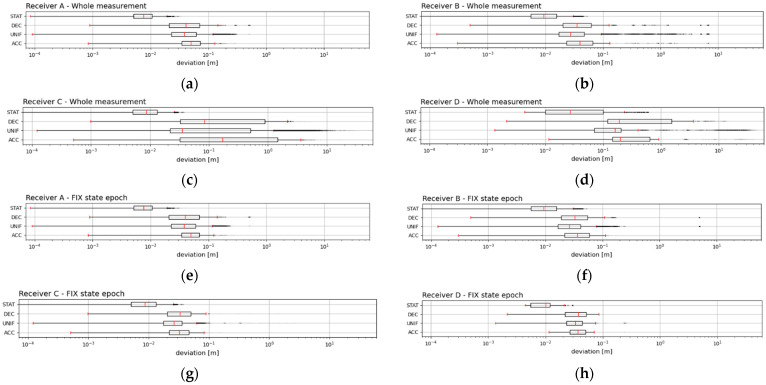
The distribution of the deviations during the current scenario phase of all the RTK receivers from the whole measurement period (**a**–**d**) and from the period “FIX” (**e**–**h**). The boxplots display plots for each condition in a rectangular area ±25% of the values occurring around the median, as shown by the red line. The extreme red lines show the boundaries dividing the contents of the external observations from the outlier area. The outsiders are shown with a black dot with a set transparency of 20%. A logarithm of base 10 was used to display the deviation axis. As expected, the best result of the median values of the positioning deviations was always the phase without movement “STAT”, and within the dynamic measurements, the best was always the “UNIF” phase of the uniform movements.

The graphs (Figure 9) below show the distribution of the positioning deviations during the individual phases of the scenario in the form of boxplots. The values of the determined median positioning deviations are also recorded in Table 7.

According to the evaluation of the deviations using the values of the medians *Me*(*STAT*), *Me*(*ACC*), *Me*(*UNIF*) and *Me*(*DEC*), it was also clear that all the RTK receivers always performed better during the “FIX” system state. The median values of the static measurement errors *Me*(*STAT*) were always significantly better than the errors of the dynamic measurement phases *Me*(*ACC*), *Me*(*UNIF*) and *Me*(*DEC*). The best accuracy in the static measurements was achieved by Receiver A, but it should be noted that this receiver provided a reference by averaging its position.

In the dynamic measurements, Receiver B was not burdened with such a large degree of influence of outliers, as was the case with the evaluation of the error indicators *µ_err_*, *s_err_* and *RMS_err_*, which was most clearly reflected in the evaluation within the period of the “FIX” system. Paradoxically, the best median values were produced by Receiver C, with Receiver B coming second, Receiver D third and Receiver A fourth. Even in this evaluation, however, the volume of the datasets for the calculation of the median values had to be taken into account, as was the case in Evaluation (3) and Evaluation (4). In the whole measurement period of the dynamic measurements, Receiver B had the best median values, Receiver A coming second, Receiver C third and Receiver D fourth.

Looking at the median values and comparing the three phases of the dynamic measurements: *Me*(*ACC*), *Me*(*UNIF*) and *Me*(*DEC*), it was clear that the best results were systematically achieved during the phases of the uniform movement “UNIF”. Therefore, it was assumed that the starting phases of the “ACC” trolley and the braking phases of the “DEC” trolley had a negative effect on the positioning capability of all the four RTK receivers. In addition, during the whole measurement period, the “ACC” phase clearly had a more negative effect on the positioning ability of all four RTK receivers than the “DEC” phase. The same was demonstrated by Receiver A and Receiver B during the “FIX” system status period.

It is clear from Table 7 that if the median values of the positioning deviations were around 10 mm in the static mode for all the monitored receivers, it was approximately four times more than in the dynamic mode. Thus, in the dynamic mode, the positioning accuracy decreased from about one centimetre to 4 centimetres, and the situation was even worse when changing the speed of the movement.

The results of the error indicators (*SSR*, *RMS_err_* and *Me*) (Table 8) were compared to each other to interpret the significance of the motions more easily to accurately measure the position of the RTK receiver. From the proportional comparison of the *SSR* indicators, it was clear that Receiver A and Receiver B measured relatively good accuracy in both the static and dynamic modes. However, even the best of the receivers (Receiver A) showed in the dynamic mode 4.8–6.4 times worse values of the error indicators (*RMS_err_* and *Me*) than in the static mode. The other receivers compared showed much worse, especially in the case of *RMS_err._*

Based on these results, it can be concluded that the errors of all the tested GNSS receivers with RTK correction in positioning were many times worse in the dynamic mode of measurements than in the static one.

Thus, as described by collective authors of reference [50], with the improvements of sensors for autonomous navigation, further improvements in tracking accuracy cannot be expected, as the proposed 1.6-cm straight-line tracking error is approximately the same as the RTK-DGPS receiver error. However, as presented in our study, the awareness of the limits given by the generally known accuracy of RTK receivers can be further increased by the error of RTK receivers acting in dynamic scenarios (e.g., during the just-mentioned rollovers or during the use of fast drone platforms). The same limit-causing distortion of the input point cloud data must then be considered by systems for the detection, classification and precise localisation of vegetation or, directly, fruits, as described, for example, by references [10,12,51,52]. Therefore, to accelerate the development and improve the quality of such functionalities, it is advisable to use the most accurate INS or to look for reference devices other than RTK-based technologies.

## 4. Conclusions

The comparison of RTK receivers using a newly developed method (MRA) allowed us to draw the following conclusions: (1) RTK receivers with a built-in INS (Receiver A and Receiver B) had better *SSR* results. Receiver D achieved a much worse *SSR* result during the dynamic measurements than during the static measurements. (2) The *RMS_err_* value of Receiver D from the whole measurement period was 94 times higher than *RMS_err_* value of Receiver A. These error indicators from the whole measurement period (3) always had worse results than the error indicators from the “FIX” status period. Distant deviations from this period had a significant impact on the values of the error indicators. During the dynamic measurements, none of the assessed RTK receivers reached the values of *RMS_err_* accuracy for the “FIX” mode as stated by the manufacturer. (4) During the static measurements, only two of the four receivers in the “FIX” period met the stated *RMS_err_* values by the manufacturer. The static measurement error indicators were also always several times better than the dynamic measurement error indicators. (5) It was possible to assume that the RTK receivers with an INS unit (Receiver A and Receiver B) were able to return to the “FIX” state earlier. (6) A linear dependence between the current values of the deviation, speed, acceleration or the state of the RTK receiver was not confirmed. (7) The median values of the deviations always had better results in the “FIX” state than in the “FLOAT” state, and in the same vein, the devices performed better in the “FLOAT” state than in the “GNSS” state. (8) The median values of the deviations always had better results in the uniform movement phase of the testing scenario than during the acceleration or braking phases. These findings should be considered in the future development of modern agricultural machinery and technology.

## Figures and Tables

**Table 1:** Essential properties of the four RTK receivers selected for evaluation. Receiver A allowed for the application of two antenna solutions to determine the absolute orientation, which was not, however, evaluated in this study. Receiver A and Receiver B contained INS. All RTK receivers were multi-GNSS types. Receiver A, Receiver B and Receiver C were multifrequency types and Receiver D a single-frequency type. The precision of the location determination in the GNSS status presented using *RMS_err_* was not indicated by the manufacturer of Receiver C. The manufacturer of Receiver D stated a higher error rate of *RMS_err_* than the other RTK receivers.

Label	Receiver A	Receiver B	Receiver C	Receiver D
Manufacturer	Novatel	Tersus	Ashtech	u-blox
Model	PwrPak7.	BX305	MB800	C94-M8P
Dynamic mode	“LAND”	default	“Automobile”	“Automotive”
Firmware	VER 7.07.01	V2.1-20180725	2.06.S850Kn27	FW3.01 HPG1.40
INS	Epson G320N IMU	6DOF IMU	-	-
GNSS antenna quantity	2	1	1	1
Signal tracking:				
GPS	L1 C/A, L1C, L2C, L2P, L5	L1, L2	L1 C/A, L1P, L2P, L2C, L5	L1 C/A
GLONASS	L1 C/A, L2C, L2P, L3, L5	L1	L1 C/A, L2 C/A	L1OF
BDS (unused)	B1, B2, B3	B1, B3	-	B1
Manufacturer *RMS_err_*:				
GNSS	1.2 m	1.5 m	-	2.5 m
RTK	0.01 m + 1 ppm	0.01 m + 1 ppm	0.01 m + 1 ppm	0.025 m + 1 ppm

**Table 2:** Error indicators during the dynamic measurements: the number of samples *n_w_* for each RTK receiver from the whole measurement period to which a valid MRA reference coordinate could be assigned (-); the number of samples *n_f_* marked as “FIX” in the NMEA message (-); *SSR* indicator indicating the RTK receiver’s ability to solve the ambiguity of the integer carrier phase during the whole measurement period (%) and the error indicators *µ_err_*, *σ_err_* and *RMS_err_* separately from the whole measurement period and separately from the “FIX” period (m).

RTK Receiver	Whole Measurement						“FIX” State Epoch		
	*n_w_*	*n_f_*	*SSR*	*µ_err_*	*s_err_*	*RMS_err_*	*µ_err_*	*s_err_*	*RMS_err_*
Receiver A	106 347	103 091	96.94%	0.047 m	0.194 m	0.06 m	0.045 m	0.182 m	0.056 m
Receiver B	106 347	97 075	91.28%	0.167 m	0.893 m	0.816 m	0.055 m	0.576 m	0.336 m
Receiver C	104 939	66 841	63.7%	0.589 m	1.418 m	2.097 m	0.029 m	0.172 m	0.041 m
Receiver D	26 150	3 600	13.77%	1.722 m	2.314 m	5.628 m	0.036 m	0.17 m	0.046 m

**Table 3:** Error indicators during the static measurements: the number of samples *n_w_* for each RTK receiver from the whole measurement period to which a valid MRA reference coordinate could be assigned (-); the number of samples *n_f_* marked as “FIX” in the NMEA message (-); *SSR* indicator indicating the RTK receiver’s ability to solve the ambiguity of the integer carrier phase during the whole measurement period (%) and the error indicators *µ_err_*, *σ_err_* and *RMS_err_* separately from the whole measurement period and separately from the “FIX” period (m).

RTK Receiver	Whole Measurement						“FIX” State Epoch		
	*n_w_*	*n_f_*	*SSR*	*µ_err_*	*s_err_*	*RMS_err_*	*µ_err_*	*s_err_*	*RMS_err_*
Receiver A	95 961	95 900	99.94%	0.008 m	0.067 m	0.009 m	0.008 m	0.067 m	0.009 m
Receiver B	92 978	92 978	100.00%	0.011 m	0.088 m	0.014 m	0.011 m	0.088 m	0.014 m
Receiver C	93 018	93 018	100.00%	0.01 m	0.076 m	0.011 m	0.01 m	0.076 m	0.011 m
Receiver D	23 267	8 971	38.56%	0.068 m	0.317 m	0.121 m	0.01 m	0.072 m	0.012 m

**Table 4:** Number of outages and recovery time of the “FIX” state after its loss: *n_o_* number of detected outages from the “FIX” state (-), *min*(*t_o_*) the shortest duration of the outage (s), *max*(*t_o_*) the longest duration of the outage (s), *µ*(*t_o_*) the mean duration of the outage (s) and *Me*(*t_o_*) the median duration of the outage (s). It was possibly assumed that the RTK receivers with an INS unit (Receiver A and Receiver B) were able to return to the “FIX” state earlier than the receivers without an INS unit (Receiver C and Receiver D).

RTK Receiver	*n_d_*	*min*(*t_d_*)	*max*(*t_d_*)	*µ*(*t_d_*)	*Me*(*t_d_*)
Receiver A	70	1 s	186 s	5.04 s	1 s
Receiver B	54	0.3 s	228.3 s	11.42 s	3.4 s
Receiver C	32	0.05 s	753.15 s	60.98 s	10.6 s
Receiver D	56	5.8 s	472 s	48.85 s	21.4 s

**Table 5:** Linear dependences of the positioning deviations with instantaneous speed, positioning deviations with instantaneous contact acceleration, current system state of a RTK receiver with instantaneous speed, current system state of a RTK receiver with instantaneous acceleration and current system state of a RTK receiver with a positioning deviation expressed by a Pearson correlation coefficient (*α* = 0.05). The current state of the RTK receivers was evaluated by three states on a linear scale with specific values: “FIX” = 2, “FLOAT” = 1 and “GNSS” = 0. All the detected dependences cannot be considered statistically significant.

RTK Receiver	Whole Measurement					“FIX” State Epoch	
	***r_dv_***	***r_da_***	***r_sv_***	***r_sa_***	***r_sd_***	***r_dv_***	***r_da_***
Receiver A	0.150	0.071	0.020	0.005	−0.358	0.190	0.079
Receiver B	0.120	0.021	−0.072	−0.014	−0.372	0.045	0.042
Receiver C	0.310	0.060	−0.586	−0.121	−0.435	0.082	0.041
Receiver D	0.360	0.005	−0.138	−0.026	−0.178	0.26	−0.032

**Table 6:** Median values of the positioning deviations divided according to the current system state of each of the four RTK receivers (m) for static and dynamic measurements. In the dynamic measurements, only Receiver A reached the “FIX” and “GNSS” states during the whole measurement period. In the static measurements, all the RTK receivers did not reach some states. For all the RTK receivers, the current system state had a significant effect on the positioning deviation, except for Receiver A in the static measurements, where a short-term condition out of “FIX” did not affect the accuracy.

RTK Receiver	Static Measurement			Dynamic Measurement		
	*Me*(*FIX*)	*Me*(*FLOAT*)	*Me*(*GNSS*)	*Me*(*FIX*)	*Me*(*FLOAT*)	*Me*(*GNSS*)
Receiver A	0.008 m	-	0.006 m	0.038 m	-	0.109 m
Receiver B	0.009 m	-	-	0.026 m	0.081 m	0.663 m
Receiver C	0.009 m	-	-	0.026 m	0.967 m	2.868 m
Receiver D	0.01 m	0.072 m	-	0.033 m	0.174 m	11.079 m

**Table 7:** Median values of the positioning deviations divided according to the phase of the scenario of the four RTK receivers (m). This evaluation was performed independently from the whole measurement period and independently from the “FIX” system status period. As expected, the best result of the median values of the positioning deviations was always the phase without movement “STAT”, and within the dynamic measurements, the best was always the “UNIF” phase of the uniform movements.

RTK Receiver	Whole Measurement				“FIX” State Epoch			
	*Me*(*STAT*)	*Me*(*ACC*)	*Me*(*UNIF*)	*Me*(*DEC*)	*Me*(*STAT*)	*Me*(*ACC*)	*Me*(*UNIF*)	*Me*(*DEC*)
Receiver A	0.008 m	0.050 m	0.038 m	0.04 m	0.008 m	0.049 m	0.037 m	0.039 m
Receiver B	0.009 m	0.039 m	0.027 m	0.035 m	0.009 m	0.036 m	0.026 m	0.032 m
Receiver C	0.009 m	0.17 m	0.035 m	0.083 m	0.009 m	0.032 m	0.026 m	0.032 m
Receiver D	0.027 m	0.198 m	0.161 m	0.187 m	0.01 m	0.036 m	0.032 m	0.037 m

**Table 8:** Proportional comparisons of the error indicators of the dynamic measurements compared to the static measurements. The RTK receivers with build-in INS (Receiver A and Receiver B) had similar results for *SSR* in the static and dynamic measurements. All four RTK receivers mostly had better error indicators (*RMS_err_* and *Me*) during the static measurements than in the dynamic measurements.

RTK Receiver	Whole Measurement			“FIX” State Epoch	
	$\frac{SSR_{\left(dyn\right)}}{SSR_{\left(stat\right)}}$	$\frac{RMS_{err\left(dyn\right)}}{RMS_{err\left(stat\right)}}$	$\frac{Me{\left(Whole\right)}_{\left(dyn\right)}}{Me{\left(Whole\right)}_{\left(\mathrm{stat}\right)}}$	$\frac{{\mathrm{RMS}}_{\mathrm{err}\left(\mathrm{dyn}\right)}}{{\mathrm{RMS}}_{\mathrm{err}\left(\mathrm{stat}\right)}}$	$\frac{\mathrm{Me}{\left(\mathrm{FIX}\right)}_{\left(\mathrm{dyn}\right)}}{\mathrm{Me}{\left(\mathrm{FIX}\right)}_{\left(\mathrm{stat}\right)}}$
Receiver A	0.97	6.4	4.9	5.9	4.8
Receiver B	0.91	59.6	3.1	24.5	2.9
Receiver C	0.64	184.9	4.1	3.6	2.9
Receiver D	0.36	46.4	6.1	4.0	3.3

## Abbreviations

AHRS	Attitude and Heading Reference System
AMSL	Above Mean Sea Level
BDS	BeiDou Navigation Satellite System
CAN	Controller Area Network
ENU	East-North-Up coordinate system
FAO	Food and Agriculture Organization
FMIS	Farm Management Information Systems
GGA	Format of essential position data sent via serial port
GLONASS	Russia’s Global Navigation Satellite System
GNSS	Global Navigation Satellite System (in general)
GPS	United States’ Global Positioning System
INS	Inertial Navigation System
LiDAR	Light Detection and Ranging
MEMS	Microelectromechanical Systems
MRA	Measurement Robotic Arm
NMEA	Serial communication standard used for GNSS output logging
NRTK	Network Real Time Kinematic
NTRIP	Networked Transport of RTCM via Internet Protocol
ppm	Parts Per Million
PPP	Precise Point Positioning
PPS	Pulse Per Second
RMS	Root Mean Square
RTCM	Serial communication standard for RTK correction signal
RTK	Real-time Kinematic
SBAS	Satellite-based Augmentation Systems
SSR	System’s ability to solve the problem of carrier phase ambiguity
UAV	Unmanned Aerial Vehicle
UTC	Coordinated Universal Time
VRS	Virtual Reference Station
ACC	Period when the trolley arm was accelerated
DEC	Period when the trolley arm was braked
FIX	Period of a known integer value of the carrier phase
FLOAT	Period of known only in a decimal of carrier phase value
GNSS	Period when the RTK receiver was not able to process the correction signal
STAT	Period when the trolley did not move
UNIF	Period when the trolley arm performed uniform movement
